# Diagnosis and Management of Hemochromatosis: 2011 Practice Guideline by the American Association for the Study of Liver Diseases

**DOI:** 10.1002/hep.24330

**Published:** 2011-07

**Authors:** Bruce R Bacon, Paul C Adams, Kris V Kowdley, Lawrie W Powell, Anthony S Tavill

**Affiliations:** 1 Division of Gastroenterology and Hepatology, Saint Louis University School of MedicineSaint Louis, MO; 2 Department of Medicine, University of Western Ontario, London Health Sciences CentreLondon, Ontario, Canada; 3 Center for Liver Disease, Virginia Mason Medical CenterSeattle, WA; 4 Royal Brisbane Hospital, University of Queensland Centre for Clinical ResearchBrisbane, Australia; 5 Department of Gastroenterology, Case Western Reserve University, and Department of Gastroenterology and Hepatology, The Cleveland ClinicCleveland, OH

This guideline has been approved by the American Association for the Study of Liver Diseases (AASLD) and represents the position of the association.

## Preamble

These recommendations provide a data-supported approach to establishing guidelines. They are based on the following: (1) a formal review and analysis of the recently published world literature on the topic; (2) the American College of Physicians *Manual for Assessing Health Practices and Designing Practice Guidelines*^1^; (3) guideline policies including the AASLD Policy on the *Development and Use of Practice Guidelines* and the American Gastroenterological Association's *Policy Statement on the Use of Medical Practice Guidelines*^2^; and (4) the experience of the authors in regard to hemochromatosis.

To more fully characterize the available evidence supporting the recommendations, the AASLD Practice Guidelines Committee has adopted the classification used by the Grading of Recommendation Assessment, Development, and Evaluation (GRADE) workgroup with minor modifications (Table 1).^3^ The strength of recommendations in the GRADE system are classified as strong (class 1) or weak (class 2). The quality of evidence supporting strong or weak recommendations is designated by one of three levels: high (level A), moderate (level B), or low-quality (level C).

**Table 1:** Grading of Recommendations, Assessment, Development, and Evaluation (GRADE)

**Strength of Recommendation**	**Criteria**
Strong (1)	Factors influencing the strength of the recommendation included the quality of the evidence, presumed patient-important outcomes, and cost
Weak (2)	Variability in preferences and values, or more uncertainty. Recommendation is made with less certainty, or higher cost or resource consumption

**Quality of Evidence**	**Criteria**
High (A)	Further research is unlikely to change confidence in the estimate of the clinical effect
Moderate (B)	Further research may change confidence in the estimate of the clinical effect
Low (C)	Further research is very likely to impact confidence on the estimate of clinical effect

Intended for use by physicians, these recommendations suggest preferred approaches to the diagnostic, therapeutic, and preventive aspects of care. They are intended to be flexible in contrast to standards of care, which are inflexible policies to be followed in every case. Specific recommendations are based on relevant published information.^3^,^4^

## Introduction

**Fig. 1:**
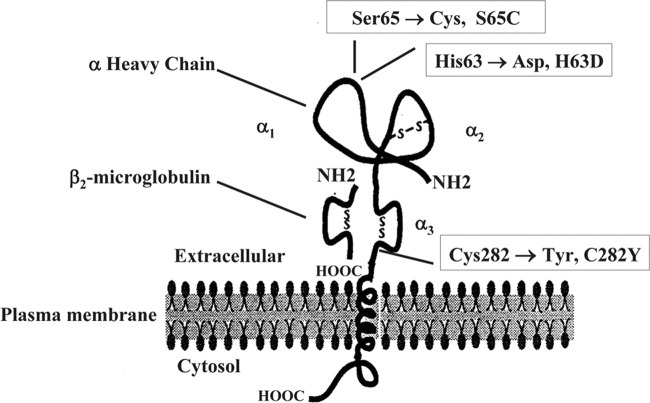
Schematic representation of the protein product of *HFE*. Most of the protein is extracellular. There is a short cytoplasmic tail and three extracellular alpha loops. The three principal mutations are identified.

Hereditary hemochromatosis (HH) remains the most common, identified, genetic disorder in Caucasians. Although its geographic distribution is worldwide, it is seen most commonly in populations of northern European origin, particularly Nordic or Celtic ancestry, in which it occurs with a prevalence of approximately 1 per 220-250 individuals.^5^,^6^ The pathophysiologic predisposition to increased, inappropriate absorption of dietary iron may lead to the development of life-threatening complications of cirrhosis, hepatocellular carcinoma (HCC), diabetes, and heart disease. The principal *HFE* gene defect was first described in 1996, and is a G-to-A missense mutation leading to the substitution of tyrosine for cysteine at amino acid position 282 of the protein product (C282Y).^7^ C282Y homozygotes account for 80%-85% of typical patients with HH.^8^ There are two other regularly identified mutations, one in which aspartate is substituted for histidine at amino acid position 63 (H63D), and the other in which cysteine is substituted for serine at amino acid position 65 (S65C). These are generally not associated with iron loading unless seen with C282Y as a compound heterozygote, C282Y/H63D or C282Y/S65C (Fig. 1). Over the last 10 years, mutations of other genes coding for iron regulatory proteins have been implicated in inherited iron overload syndromes (e.g., hepcidin, hemojuvelin, transferrin receptor 2, and ferroportin). These are thought to account for most of the non-*HFE* forms of HH.^9^

With the advent of genetic testing in the late 1990s, *HFE*-related HH is now frequently identified in asymptomatic probands and in presymptomatic relatives of patients who are known to have the disease. Accordingly, a genetic diagnosis can be applied to individuals who have not yet developed any phenotypic expression. Therefore, these individuals have a “genetic susceptibility” to developing iron overload but may never do so, for reasons that are still to be determined.^6^,^10^–^12^ This observation has changed the way we think about hemochromatosis. Twenty years ago, it was considered that all individuals who were genetically susceptible would ultimately have evidence of phenotypic expression. Now, it is clear that phenotypic expression only occurs in approximately 70% of C282Y homozygotes, and fewer than 10% of C282Y homozygotes will develop severe iron overload accompanied by organ damage and clinical manifestations of hemochromatosis.^10^,^12^ This acknowledgment has led to a recognition of the different stages and progression of hemochromatosis identified at a consensus conference of the European Association for the Study of Liver Diseases in 2000.^13^ These stages are defined as follows:

- Stage 1 refers to those patients with the genetic disorder with no increase in iron stores who have “genetic susceptibility.”
- Stage 2 refers to those patients with the genetic disorder who have phenotypic evidence of iron overload but who are without tissue or organ damage.
- Stage 3 refers to those individuals who have the genetic disorder with iron overload and have iron deposition to the degree that tissue and organ damage occurs.

This organizational schema is important to allow clinicians to categorize patients who have positive genetic test results.

## Causes of Iron Overload

The current classification of iron overload syndromes divides patients into three groups (Table 2): (1) those who have inherited causes of iron overload, (2) those who have various causes of secondary iron overload, and (3) a small miscellaneous group. Approximately 85%-90% of patients who have inherited forms of iron overload are homozygous for the C282Y mutation in *HFE*, with a small minority who are compound heterozygotes, meaning that one allele has the C282Y mutation and one allele has the H63D or the S65C mutation. The remaining 10%-15% of patients who have inherited forms of iron overload most likely have mutations in one of the other aforementioned genes involved in iron homeostasis.^9^ Causes of secondary iron overload are divided between those causes related to iron loading anemias, those related to chronic liver disease, transfusional iron overload, and miscellaneous causes. Oral iron ingestion does not lead to iron overload except in genetically predisposed individuals or those who have ineffective erythropoiesis.

**Table 2:** Classification of Iron Overload Syndromes

Hereditary Hemochromatosis
*HFE*-related
C282Y/C282Y
C282Y/H63D
Other *HFE* mutations
Non–*HFE*-related
Hemojuvelin (*HJV*)
Transferrin receptor-2 (*TfR2*)
Ferroportin (*SLC40A1*)
Hepcidin (*HAMP*)
African iron overload
Secondary Iron Overload
Iron-loading anemias
Thalassemia major
Sideroblastic
Chronic hemolytic anemia
Aplastic anemia
Pyruvate kinase deficiency
Pyridoxine-responsive anemia
Parenteral iron overload
Red blood cell transfusions
Iron–dextran injections
Long-term hemodialysis
Chronic liver disease
Porphyria cutanea tarda
Hepatitis C
Hepatitis B
Alcoholic liver disease
Nonalcoholic fatty liver disease
Following portocaval shunt
Dysmetabolic iron overload syndrome
Miscellaneous
Neonatal iron overload
Aceruloplasminemia
Congenital atransferrinemia

Other inherited forms of iron overload, classified as non–*HFE*-related HH, are juvenile hemochromatosis and iron overload resulting from mutations in the genes for transferrin receptor 2 (*TfR2*), or ferroportin (*SLC40A1*).^9^ Juvenile HH is characterized by rapid iron accumulation. Mutations in two different genes (hemojuvelin and hepcidin) have been shown to cause two forms of juvenile HH.^14^ The more common mutation occurs in the hemojuvelin (*HJV*) gene on chromosome 1q.^15^ Mutations in the hepcidin gene (*HAMP*) also produce a form of juvenile HH, but this is much less common.^14^ Hepcidin is a 25–amino acid peptide produced in the liver that down-regulates iron absorption. Mutations in the *TfR2* gene produce an autosomal recessive form of HH that is clinically similar to *HFE*-related HH.^16^ These mutations may cause abnormal iron sensing by hepatocytes, which is the predominant site of TfR2 expression. The distribution of excess iron is similar to that in *HFE*-related HH, namely, primarily in hepatic parenchymal cells.^16^ A rare autosomal dominant form of HH results from two categories of mutations in the gene for the iron transporter protein, ferroportin. “Loss-of-function” mutations decrease the cell surface localization of ferroportin, thereby reducing its ability to export iron.^17^,^18^ The result is iron deposition primarily in macrophages, and this disorder is called “ferroportin disease”. The second category of mutation includes “gain-of-function” ferroportin mutations that abolish hepcidin-induced ferroportin internalization and degradation^18^; distribution of iron is similar to *HFE*-related HH, concentrating predominantly in parenchymal cells.

African iron overload occurs primarily in sub-Saharan Africa and is now considered to be the result of a non–*HFE*-related genetic abnormality that can be exacerbated by dietary iron loading.^19^ Some individuals with African iron overload drink an iron-rich fermented beverage, but iron overload can also occur in people who do not drink this beverage.^19^

### Causes of Secondary Iron Overload and Miscellaneous Disorders

Individuals who absorb excessive amounts of iron as a result of an underlying defect other than any of the previously mentioned inherited disorders have secondary iron overload.^20^ The most common causes of secondary iron overload are individuals with ineffective erythropoiesis, parenteral iron overload, and liver disease. Individuals who receive blood transfusions and who have transfusional or parenteral iron overload should be distinguished from those who have other causes of secondary iron overload. Parenteral iron overload is always iatrogenic, in that blood or iron (given parenterally) must be ordered by a health care provider prior to its administration. Many individuals with ineffective erythropoiesis who have decreased utilization of iron by the bone marrow also have transfusional iron overload because of a requirement for transfusions.^20^

Recently, it has been found that neonatal hemochromatosis is actually a form of congenital alloimmune hepatitis with subsequent iron deposition.^21^ In these cases, immune-mediated liver injury in the fetus is associated with the development of iron overload. Administration of intravenous immunoglobulin during pregnancy slows or prevents the development of this condition.^22^ Other rare miscellaneous disorders include congenital atransferrinemia and aceruloplasminemia.

## Pathophysiology

There are four main categories of pathophysiological mechanisms of HH that should be mentioned: (1) the increased absorption of dietary iron in the upper intestine, (2) decreased expression of the iron-regulatory hormone hepcidin, (3) the altered function of HFE protein, and (4) tissue injury and fibrogenesis induced by iron.

### Intestinal Iron Absorption

The first link between HFE protein and cellular iron metabolism resulted from the observation that the HFE protein along with β_2_-microglobulin forms a complex with transferrin receptor-1 (TfR1).^23^ This physical association was observed in cultured cells and in duodenal crypt enterocytes, which have been considered to be the predominant site of regulation of dietary iron absorption. The observation that HFE protein and TfR1 were physically associated led to a number of investigations of the effect of HFE protein on TfR1-mediated iron uptake and cellular iron stores.^24^ The “crypt cell hypothesis” of iron regulation is now regarded as much less important since the discovery of the central role of hepcidin in the regulation of iron metabolism.

### Hepcidin

Hepcidin is a 25–amino acid peptide that influences systemic iron status.^25^ It is considered to be the principal iron-regulatory hormone. Alteration in the regulation of hepcidin plays an important role in the pathogenesis of hemochromatosis. Hepcidin is expressed predominantly in hepatocytes and is secreted into the circulation. It binds to ferroportin, which is found in macrophages and on the basolateral surface of enteroctyes. When hepcidin binds to ferroportin, the ferroportin is internalized and degraded and iron export by these two cell types (macrophages and enterocytes) is inhibited.^26^ Hepcidin expression induced by excess iron or inflammation results in decreased intestinal iron absorption and diminished iron release from macrophages.^25^

In contrast, hepcidin expression is decreased by iron deficiency, ineffective erythropoiesis, and hypoxia, with resulting increases in iron absorption from the intestine and release of iron from macrophages.^25^ Mutations in human disease or murine knockouts of the genes for HFE, hemojuvelin, hepcidin, or TfR2 decrease hepcidin expression with a resulting increase in intestinal iron absorption via up-regulation of ferroportin levels in enterocytes.^25^

Studies have revealed that iron-induced regulation of hepcidin expression involves a bone morphogenetic protein 6 (BMP6)-dependent signaling pathway.^27^ BMP6 binds to a specific receptor on hepatocytes triggering SMAD protein–dependent activation of hepcidin expression. Selective inhibition of BMP6 signaling abrogates iron-induced up-regulation of hepcidin.^27^ Hemojuvelin is a BMP6 coreceptor, and it facilitates the binding of BMP6 to its receptor; knockout of the HJV gene markedly decreases BMP6 signaling in hepcidin expression and causes iron overload.^28^

### HFE Protein

**Fig. 2:**
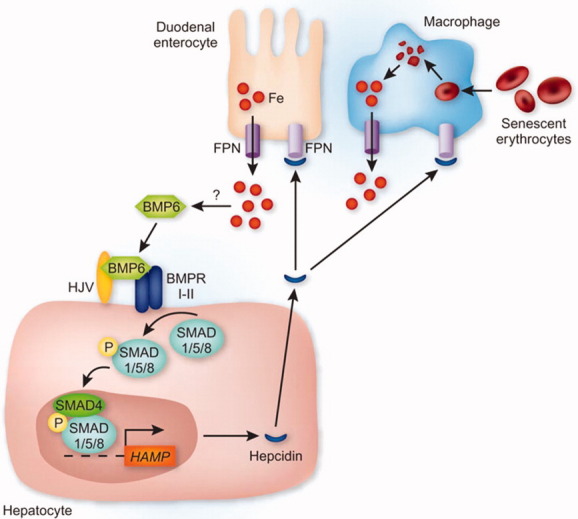
Summary of interactions between duodenal enterocytes, hepatocytes, and macrophages in iron homeostasis regulated by hepcidin. FPN, ferroportin. (Adapted from Camaschella C. BMP6 orchestrates iron metabolism. Nat Genet 2009;41:386–388. Used with permission from *Nature Genetics*. Copyright © 2009, Nature Publishing Group).

The extracellular domain of HFE protein consists of three loops with intramolecular disulfide bonds within the second and third loops^7^ (Fig. 1). The structure of the HFE protein is similar to that of other major histocompatibility complex class-1 proteins, but evidence indicates that HFE protein does not participate in antigen presentation.^29^ HFE protein is physically associated with β_2_-microglobulin, similar to other major histocompatibility complex class-1 molecules. The major mechanisms by which HFE influences iron-dependent regulation of hepcidin remain unclear. HFE can bind to both TfR2 and to the classic transferrin receptor TfR1.^23^,^30^ In addition, both HFE and TfR2 may interact with HJV, suggesting that a complex of HFE and TfR2 may play a regulatory role in BMP6 signaling.^28^ One proposed explanation suggests that the complex of TfR1 and HFE acts as an iron sensor at the cell membrane of the hepatocyte.^30^ As transferrin saturation (TS) increases, diferric transferrin displaces HFE from TfR1, thereby making HFE available to bind to TfR2. It is postulated that the complex of HFE and TfR2 then influences hepcidin expression. Figure 2 summarizes these interactions.^31^

### Liver Damage

Another major pathophysiologic mechanism in HH relates to the liver damage that results from iron overload.^32^ In patients with advanced HH, hepatic fibrosis and cirrhosis are the principal pathological findings. Numerous studies using experimental hepatic iron overload have identified iron-dependent oxidative damage and associated impairment of membrane-dependent functions of mitochondria, microsomes, and lysosomes.^33^,^34^ One hypothesis is that iron-induced lipid peroxidation occurs in hepatocytes and causes hepatocellular injury or death. Kupffer cells become activated byproducts released from injured iron-loaded hepatocytes and produce profibrogenic cytokines, which in turn stimulate hepatic stellate cells to synthesize increased amounts of collagen, thereby leading to pathologic fibrosis.^32^,^35^

## Clinical Features

Hemochromatosis is increasingly being recognized by clinicians. Nonetheless, it is still underdiagnosed, because it is often considered a rare disorder that is manifested by the clinical findings seen in fully established disease consisting of cirrhosis, diabetes, and skin pigmentation (so-called “bronze diabetes”). Genetic susceptibility (C282Y homozygosity) for hemochromatosis is seen in approximately one in 250 Caucasians; however, fully expressed disease with end-organ manifestations is seen in fewer than 10% of these individuals.^10^,^12^ The reasons for the lack of phenotypic expression are unknown. It may involve interactions with gene products of other proteins involved in iron homeostasis (with or without mutation). This can explain the discrepancy between the high incidence of C282Y homozygosity in Caucasians (one in 250) versus how infrequently the full clinical manifestations of the disease are seen (approximately one in 2500). The heterozygote genotype (C282Y/wild type) is found in approximately one in 10 individuals and may be associated with elevated serum iron markers, but without associated tissue iron overload or damage.

Clinical manifestations in patients reported in series from the 1950s to the 1980s showed that most reported patients had classic symptoms and findings of advanced hemochromatosis (Table 3).^36^–^38^ By the 1990s, HH was increasingly being identified in patients who had abnormal iron studies on routine chemistry panels or by patients having been identified by family screening.^39^,^40^ When patients with HH were identified in this way, approximately 75% of them did not have symptoms and did not exhibit any of the end-stage manifestations of the disease. Currently, in large population screening studies, only approximately 70% of C282Y homozygotes are found to have an elevated ferritin level indicative of increased iron stores (Table 4), and only a small percentage of these patients have clinical consequences of iron storage disease.^6^,^10^,^12^,^41^,^42^ More men than women have increased ferritin levels. Nonetheless, it is still important for clinicians to be aware of the symptoms that patients may exhibit and the physical findings with which they can present.

**Table 3:** Principal Clinical Features in Hereditary Hemochromatosis

	Study (Year)				
	
Features	Milder et al.^37^ (1980)	Edwards et al.^36^ (1980)	Niederau et al.^38^ (1985)	Adams et al.^39^ (1991)	Bacon and Sadiq^40^ (1997)
Number of subjects	34†	35*	163*	37‡	40
*Symptoms* (%)					
Weakness, lethargy	73	20	83	19	25
Abdominal pain	50	23	58	3	0
Arthralgias	47	57	43	40	13
Loss of libido, impotence	56	29	38	32	12
Cardiac failure symptoms	35	0	15	3	0
*Physical and Diagnostic Findings* (%)					
Cirrhosis (biopsy)	94	57	69	3	13
Hepatomegaly	76	54	83	3	13
Splenomegaly	38	40	13	–	–
Loss of body hair	32	6	20	–	–
Gynecomastia	12	–	8	–	–
Testicular atrophy	50	14	–	–	–
Skin pigmentation	82	43	75	9	5
Clinical diabetes	53	6	55	11	–

* Patient selection occurred by both clinical features and family screening.

† Only symptomatic index cases were studied.

‡ Discovered by family studies.

**Table 4:** Prevalence of C282Y Homozygotes Without Iron Overload in Large Screening Studies

Population Sample	Country	*n*	Prevalence of Homozygotes	C282Y Homozygotes with Normal Ferritin Level (%)
Primary care (12)	USA	41,038	1 in 270	35
General public (11)	Norway	65,238	1 in 220	13
Primary care (6)	North America	99,711	1 in 333	31
General public (10)	Australia	29,676	1 in 146	32
Total		235,663	1 in 240	30

When patients present with symptoms, hemochromatosis should be considered when there are complaints of fatigue, right upper quadrant abdominal pain, arthralgias, (typically of the second and third metacarpophalangeal joints), chondrocalcinosis, impotence, decreased libido, and symptoms of heart failure or diabetes (Table 5). Similarly, physical findings of an enlarged liver, particularly in the presence of cirrhosis, extrahepatic manifestations of chronic liver disease, testicular atrophy, congestive heart failure, skin pigmentation, changes of porphyria cutanea tarda (PCT), or arthritis should raise the suspicion of hemochromatosis (Table 6). Many of these features are indicative of disease processes other than hemochromatosis, but the thoughtful clinician will make sure that hemochromatosis has been considered when patients who exhibit these symptoms or signs are seen. Currently, most new patients with HH come to medical attention because of screening, such as in family studies, or by evaluation of abnormal laboratory studies by primary care physicians. In older series of patients with HH, when patients were identified by symptoms or physical findings of the disease, women typically presented approximately 10 years later than men, and there were approximately 10 times as many men presenting as women. This sex difference is likely because of menstrual blood loss and maternal iron loss during pregnancy having a “protective” effect for women. More recently, with a greater proportion of patients identified by screening studies, the age of diagnosis for women and men has equalized, and the numbers of men and women identified are roughly equivalent.^6^,^10^ Nonetheless, the proportion of C282Y homozygous women with definite disease manifestations (e.g., liver disease, arthritis) is significantly lower than men (1% versus 25%, respectively).^10^

**Table 5:** Symptoms in Patients with HH

Asymptomatic
Abnormal serum iron studies on routine screening chemistry panel
Evaluation of abnormal liver tests
Identified by family screening
Nonspecific, systemic symptoms
Weakness
Fatigue
Lethargy
Apathy
Weight loss
Specific, organ-related symptoms
Abdominal pain (hepatomegaly)
Arthralgias (arthritis)
Diabetes (pancreas)
Amenorrhea (cirrhosis)
Loss of libido, impotence (pituitary, cirrhosis)
Congestive heart failure (heart)
Arrhythmias (heart)

**Table 6:** Physical Findings in Patients with HH

Asymptomatic
No physical findings
Hepatomegaly
Symptomatic
Liver
Hepatomegaly
Cutaneous stigmata of chronic liver disease
Splenomegaly
Liver failure: ascites, encephalopathy, and associated features
Joints
Arthritis
Joint swelling
Chondrocalcinosis
Heart
Dilated cardiomyopathy
Congestive heart failure
Skin
Increased pigmentation
Porphyria cutanea tarda
Endocrine
Testicular atrophy
Hypogonadism
Hypothyroidism

***Recommendations:***

***1. We recommend that patients with abnormal iron studies should be evaluated as patients with hemochromatosis, even in the absence of symptoms. (A)***

***2. All patients with evidence of liver disease should be evaluated for hemochromatosis. (1B*)**

## Diagnosis

**Fig. 3:**
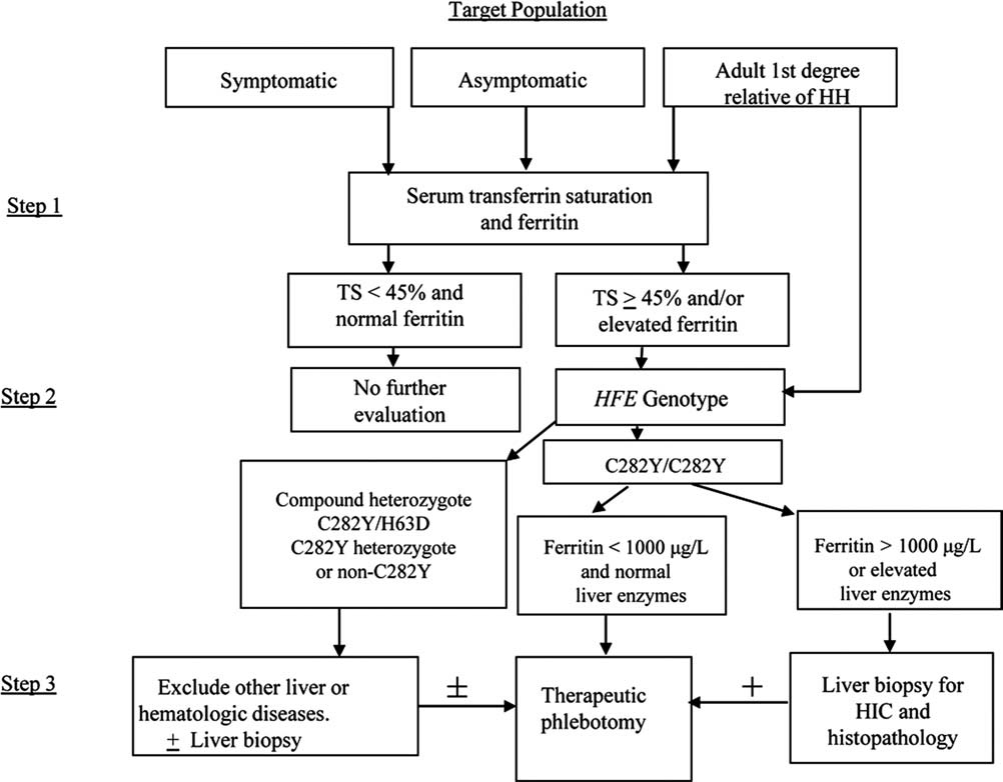
An algorithm can provide some further direction regarding testing and treatment for HH. The algorithm is modified from the version used in the previous AASLD guidelines.

The clinical diagnosis of hemochromatosis is based on documentation of increased iron stores, demonstrated by elevated serum ferritin levels, which reflects an increase in hepatic iron content. HH can be further defined genotypically by the familial occurrence of iron overload associated with C282Y homozygosity or C282Y/H63D compound heterozygosity. Serologic iron markers (TS, ferritin) are widely available, and the majority of patients with HH are now identified while still asymptomatic and without evidence of hepatic fibrosis or cirrhosis. There are certain high-risk groups that should be targeted for evaluation, such as those with a family history of HH, those with suspected organ involvement, and those with chance detection of biochemical and/or radiological abnormalities suggestive of the possibility of iron overload. It is generally recommended that all patients with abnormal liver function have iron studies done at some point in their evaluation. The algorithm outlined in Fig. 3 can provide some further direction regarding testing and is modified from the version used in the previous AASLD guidelines.^42^

The initial approach to diagnosis is by indirect markers of iron stores, namely TS or unsaturated iron-binding capacity and serum ferritin (Table 7). TS is calculated from the ratio of serum iron to total iron-binding capacity. In some laboratories, the total iron-binding capacity is calculated from the sum of the serum iron and the unsaturated iron-binding capacity, whereas in others, it is calculated indirectly from the transferrin concentration in the serum. A recent study, using fasting samples, has shown no improvement in sensitivity or specificity in the detection of C282Y homozygotes.^43^ Accordingly, this prior recommendation is no longer absolutely necessary, although it is advisable to confirm an elevated TS with a second determination and it is not unreasonable in our opinion to do this on a fasting specimen. Over the years, different studies have used a variety of cutoff values for TS to identify patients eligible for further testing. Although a cutoff TS value of 45% is often chosen for its high sensitivity for detecting C282Y homozygotes, it has a lower specificity and positive predictive value compared to higher cutoff values. Thus, using a cutoff TS of 45% will also identify persons with minor secondary iron overload as well as some C282Y/wild-type heterozygotes, and these cases will require further evaluation.^44^

**Table 7:** Laboratory Findings in Patients with HH

		Patients with HH	
		
Measurements	Normal Subjects	Asymptomatic	Symptomatic
Blood			
Serum iron level (μg/dL)	60-80	150-280	180-300
TS (%)	20-50	45-100	80-100
Serum ferritin level (μg/L)			
Men	20-200	150-1000	500-6000
Women	15-150	120-1000	500-6000
Liver			
Hepatic iron concentration			
μg/g dry weight	300-1500	2000-10,000	8000-30,000
μmol/g dry weight	5-27	36-179	140-550
Hepatic iron index*	<1.0	>1.9	>1.9
Liver histology			
Perls' Prussian blue stain	0-1+	2+ to 4+	3+, 4+

* Hepatic iron index is calculated by dividing the hepatic iron concentration (in μmol/g dry weight) by the age of the patient (in years). With increased knowledge of genetic testing results in patients with iron overload, the utility of the hepatic iron index has diminished.

Serum ferritin has less biological variability than TS, but it has a significant false positive rate because of elevations related to inflammation. Ferritin can be elevated in the absence of increased iron stores in patients with necroinflammatory liver disease (alcoholic liver disease [ALD], chronic hepatitis B and C, nonalcoholic fatty liver disease [NAFLD]), in lymphomas, and in patients with other nonhepatic chronic inflammatory conditions. In fact, in the general population, iron overload is not the most common cause of an elevated ferritin level. Nonetheless, in the absence of other inflammatory processes, several studies of families with HH have demonstrated that the serum ferritin concentration provides a valuable correlation with the degree of body iron stores. In most circumstances, serum ferritin provides additional confirmation of the significance of an elevated TS in C282Y homozygotes. In a study of individuals <35 years of age, serum ferritin in the normal range in combination with a TS < 45% had a negative predictive value of 97% for excluding iron overload.^45^ In a large study correlating phenotypic and genotypic markers in a primary care population in California, a serum ferritin >250 μg/L in men and >200 μg/L in women was positive in 77% and 56%, respectively, of C282Y homozygotes.^12^ In the HEIRS (HEmochromatosis and IRon Overload Screening) study that screened 99,711 North American participants, serum ferritin levels were elevated (>300 μg/L in men, >200 μg/L in women) in 57% of female and 88% of male C282Y homozygotes.^6^ It is recognized that a variety of disease conditions unrelated to iron overload may cause a nonspecific rise in serum ferritin, and in the absence of an elevated TS, this rise may be nonspecific. Conversely, iron overload may be present in a patient with an elevated ferritin and a normal TS, particularly in non–*HFE*-related iron overload or in a C282Y/H63D compound heterozygote.^46^

Serum ferritin levels have an additional value as a predictor of advanced fibrosis and cirrhosis in confirmed HH. Several studies have demonstrated that a level of serum ferritin <1000 μg/L is an accurate predictor for the absence of cirrhosis, independent of the duration of the disease.^47^–^49^ A serum ferritin level >1000 μg/L with an elevated aminotransferase level (alanine aminotransferase [ALT] or aspartate aminotransferase [AST]) and a platelet count <200 × 10^9^/L predicted the presence of cirrhosis in 80% of C282Y homozygotes.^50^

***Recommendations:***

***3. In a patient with suggestive symptoms, physical findings, or family history, a combination of TS and ferritin should be obtained rather than relying on a single test. (1B) If either is abnormal (TS ≥ 45% or ferritin above the upper limit of normal), then* HFE *mutation analysis should be performed. (1B*)**

***4. Diagnostic strategies using serum iron markers should target high-risk groups such as those with a family history of HH or those with suspected organ involvement. (1B*)**

## Family Screening

Once a patient with HH has been identified (proband), family screening should be recommended for all first-degree relatives. For ease of testing, both genotype (*HFE* mutation analysis) and phenotype (ferritin and TS) should be performed simultaneously at a single visit. For children of an identified proband, *HFE* testing of the other parent is generally recommended, because if results are normal, the child is an obligate heterozygote and need not undergo further testing because there is no increased risk of iron loading.^51^ If C282Y homozygosity or compound heterozygosity is found in adult relatives of a proband, and if serum ferritin levels are increased, then therapeutic phlebotomy can be initiated. If ferritin level is normal in these patients, then yearly follow-up with iron studies is indicated. When identified, C282Y heterozygotes and H63D heterozygotes can be reassured that they are not at risk for developing progressive or symptomatic iron overload. Occasional H63D homozygotes can develop mild iron overload.^52^ However, it should be recognized that any of these genotypes can be a cofactor for the development of liver disease when they occur in conjunction with other liver diseases such as PCT, hepatitis C infection, ALD, or NAFLD. Relatives who are identified as H63D heterozygotes or H63D homozygotes can be reassured that they are generally not at risk of progressive iron overload, although they may have minor abnormalities in serum iron measurements such as TS or ferritin.

Family studies have concluded that many homozygous relatives of probands demonstrate biochemical and clinical expression of disease.^53^,^54^ Furthermore, a recent population study of approximately 30,000 Caucasian subjects aged 40-69 years identified 203 C282Y homozygotes (108 females, 95 males). These subjects were evaluated sequentially over a 12-year period, prior to available knowledge of their genotype. Documented iron overload-related disease was present in 28% of males and 1% of females, especially when serum ferritin levels were >1000 μg/L.^10^

***Recommendations:***

***5. We recommend screening (iron studies and* HFE *mutation analysis) of first-degree relatives of patients with* HFE-*related HH to detect early disease and prevent complications. (1A*)**

## Liver Biopsy

Since the advent of *HFE* mutation analysis, liver biopsy has become less important as a clinical tool in the diagnosis of HH. Liver biopsy should be considered only for the purpose of determining the presence or absence of advanced fibrosis or cirrhosis, which does have prognostic value. Identification of cirrhosis may lead to adjustments in clinical management, such as screening for HCC and esophageal varices (and other features of portal hypertension).^55^ The risks of liver biopsy have been reviewed, with mild bleeding after biopsy reported to be in the range of 1%-6%, and mortality associated with a complication of less than 1:10,000.^56^

Serum ferritin levels can help identify patients who may benefit most from having a liver biopsy. Several studies have demonstrated that C282Y homozygotes with a serum ferritin >1000 μg/L are at an increased risk of cirrhosis, with a prevalence of 20%-45%.^49^,^50^ In contrast, fewer than 2% of C282Y homozygotes with a ferritin level <l000 μg/L at the time of diagnosis have cirrhosis or bridging fibrosis in the absence of another risk factor such as excessive alcohol consumption, viral hepatitis, or fatty liver disease.^47^–^50^ A recent study of more than 670 asymptomatic C282Y homozygotes described the prevalence of advanced hepatic fibrosis.^41^ In this study, a liver biopsy was performed in 350 subjects because of elevated serum ferritin levels (using a cutoff of 500 μg/L) or abnormal serum liver enzyme results, the presence of hepatomegaly, or a combination of these. The majority of these biopsies were performed for diagnosis of HH prior to the availability of *HFE* mutation analysis. Cirrhosis was present in 5.6% of all males and 1.9% of all females. All subjects with cirrhosis had a hepatic iron concentration (HIC) >200 μmol/g dry weight (approximately seven times the upper limit of normal). A serum ferritin level >1000 μg/L had 100% sensitivity and 70% specificity for identification of cirrhosis. No subject with a serum ferritin level <1000 μg/L had cirrhosis. These observations must be tempered when patients with HH also consume large amounts of alcohol. An Australian study showed that >60% of patients with HH who consumed >60 g alcohol/day had cirrhosis, compared to <7% of those who consumed less alcohol.^57^

Based on these recent studies, it can be concluded that serum ferritin is the single most important predictor of the presence of advanced hepatic fibrosis in C282Y homozygotes. Therefore, liver biopsy does not need to be performed when ferritin is <l000 μg/L, in the absence of excess alcohol consumption and elevated serum liver enzymes.

Patients with elevated serum iron studies, but who lack C282Y homozygosity, should be considered for liver biopsy if they have elevated liver enzymes or other clinical evidence of liver disease. These patients may have non-HH liver disease such as NAFLD, ALD, or chronic viral hepatitis.

When liver biopsy is performed, routine histopathologic evaluation should include standard hematoxylin–eosin and Masson's trichrome stains as well as Perls' Prussian blue stains for evaluating the degree and cellular distribution of hepatic iron stores. In addition, a portion of liver tissue can be obtained for measurement of HIC. It should be recognized that HIC can also be measured from formalin-fixed, deparaffinized specimens, but at least 4 mg dry weight of tissue should be available for evaluation.^58^ Qualitative and semiquantitative methods for grading the degree of stainable hepatic iron have been described. The Batts–Ludwig system uses an estimation of the proportion of hepatocytes that stain for iron, ranging from solely zone 1 (periportal) to inclusion of zones 2 and 3 (pericentral). The grading of iron staining ranges from grade 1 to grade 4, with grade 4 representing panlobular iron deposition.^59^ A semiquantitative “histological hepatic iron index” has been proposed based on the size and density of iron granules in hepatocytes, sinusoidal lining cells, and portal cells.^60^ This formula can be used to calculate a total iron score, and this system has been validated and found to be useful to differentiate heterozygotes from homozygotes. It is not widely used outside the research setting.

Hepatic iron index (HII) was first introduced in 1986 and was used frequently to support a diagnosis of HH when the HII was >1.9, prior to the advent of *HFE* mutation analysis.^61^,^62^ HII, which measures the rate of hepatic iron accretion, is calculated by dividing the HIC (in μmol/g) by the patient's age in years and was based on the concept that homozygotes would continue to absorb excess dietary iron throughout their lifetime, whereas those who were heterozygotes or those with iron overload due to associated alcohol use would not. Several studies showed that most homozygotes with iron overload had an HII > l.9 μmol/g/year, whereas patients with other chronic diseases had an HII < 1.9.^63^,^64^ The availability of genetic testing has now shown that phenotypic expression of homozygosity can occur at a much lower HIC and a much lower HII, and therefore the HII is no longer routinely used. Recent studies show good correlation between HIC determined on liver biopsy samples with HIC estimated by proton transverse relaxation time determined by magnetic resonance imaging.^64^

***Recommendations:***

***6. Liver biopsy is recommended to stage the degree of liver disease in C282Y homozygotes or compound heterozygotes if liver enzymes (ALT, AST) are elevated or if ferritin is >1000 μg/L. (1B*)**

## Role of Liver Biopsy in Non–*HFE*-related HH

Liver biopsy may provide both diagnostic and prognostic information in patients with iron overload who are not C282Y homozygotes. Abnormal serum iron studies are identified in approximately 50% of patients with other liver diseases such as ALD, NAFLD, or chronic viral hepatitis. Liver biopsy is used to evaluate those patients both from the standpoint of their underlying disease, determining the stage of fibrosis, and to determine the degree of iron loading. In the secondary iron overload seen with other liver diseases, iron deposition is usually mild (1+ to 2+) and generally occurs in both perisinusoidal lining cells (Kupffer cells) and in hepatocytes in a panlobular distribution.^59^ Liver biopsy is also useful to identify the different pattern of iron overload seen in patients with ferroportin disease, wherein the iron deposition is predominantly in reticuloendothelial cells or is in a mixed pattern of hepatocytes and reticuloendothelial cells without a periportal predominance.^9^

***Recommendations:***

***7. Liver biopsy is recommended for diagnosis and prognosis in patients with phenotypic markers of iron overload who are not C282Y homozygotes or compound heterozygotes. (2C*)**

***8. We recommend that in patients with non*–HFE-*related HH, data on hepatic iron concentration is useful, along with histopathologic iron staining, to determine the degree and cellular distribution of iron loading present. (2C*)**

## Treatment of Hemochromatosis

Although there has never been a randomized controlled trial of phlebotomy versus no phlebotomy in treatment of HH, there is nonetheless, evidence that initiation of phlebotomy before the development of cirrhosis and/or diabetes will significantly reduce the morbidity and mortality of HH.^65^,^66^ Therefore, early identification and preemptive treatment of those at risk is generally recommended. This includes treatment of asymptomatic individuals with homozygous HH and markers of iron overload, as well as others with evidence of increased levels of hepatic iron. In symptomatic patients, treatment is also advocated to reduce progression of organ damage. Certain clinical features are likely to be ameliorated by phlebotomy (malaise, fatigue, skin pigmentation, insulin requirements for diabetics, and abdominal pain), whereas other features are either less responsive to iron removal or do not respond at all (Table 8). These include arthropathy, hypogonadism, and advanced cirrhosis. In some cases, hepatic fibrosis and cirrhosis show regression after phlebotomy.^67^ The life-threatening complications of established cirrhosis, particularly HCC, continue to be a threat to survival even after adequate phlebotomy. Therefore, patients with cirrhosis should continue to be screened for HCC following phlebotomy. HCC accounts for approximately 30% of HH-related deaths, whereas complications of cirrhosis account for an additional 20%.^66^,^68^ HCC is exceptionally rare in noncirrhotic HH, which provides an additional argument for preventive therapy prior to the development of cirrhosis.^69^

**Table 8:** Response to Phlebotomy Treatment in Patients with HH

Reduction of tissue iron stores to normal
Improved survival if diagnosis and treatment before development of cirrhosis and diabetes
Improved sense of well-being, energy level
Improved cardiac function
Improved control of diabetes
Reduction in abdominal pain
Reduction in skin pigmentation
Normalization of elevated liver enzymes
Reversal of hepatic fibrosis (in approximately 30% of cases)
No reversal of established cirrhosis
Elimination of risk of HH-related HCC if iron removal is achieved before development of cirrhosis
Reduction in portal hypertension in patients with cirrhosis
No (or minimal) improvement in arthropathy
No reversal of testicular atrophy

Phlebotomy remains the mainstay of treatment for HH (Table 9). One unit of blood contains approximately 200-250 mg iron, depending on the hemoglobin concentration, and should be removed once or twice per week as tolerated. In patients with HH who may have total body iron stores >30 g, therapeutic phlebotomy may take up to 2-3 years to adequately reduce iron stores. Each phlebotomy should be preceded by measurement of the hematocrit or hemoglobin so as to avoid reducing the hematocrit/hemoglobin to <80% of the starting value. TS usually remains elevated until iron stores are depleted, whereas ferritin, which may initially fluctuate, eventually begins to fall progressively with iron mobilization and is reflective of depletion of iron stores. Serum ferritin analysis should be performed after every 10-12 phlebotomies (approximately 3 months) in the initial stages of treatment. It can be confidently assumed that excess iron stores have been mobilized when the serum ferritin drops to between 50 and 100 μg/L. As the target range of 50-100 μg/L is approached, testing may be repeated more frequently to preempt the development of overt iron deficiency. It is not necessary for patients to achieve iron deficiency and in fact, this should be avoided. Phlebotomy can be stopped at the point at which iron stores are depleted, and the patient should be assessed for whether they require maintenance phlebotomy. For reasons that are unclear, not all patients with HH reaccumulate iron and, accordingly, they may not need a maintenance phlebotomy regimen. Therefore, the frequency of maintenance phlebotomy varies among individuals, due to the variable rate of iron accumulation in HH. Some patients (either male or female) require maintenance phlebotomy monthly, whereas others who reaccumulate iron at a slower rate may need only 1-2 units of blood removed per year. In the United States, blood acquired by therapeutic phlebotomy may be used for blood donation in some institutions, and both the American Red Cross and the U.S. Food and Drug Administration have deemed that the blood is safe for transfusion.^70^

**Table 9:** Treatment of Hemochromatosis

Hereditary hemochromatosis
One phlebotomy (removal of 500 mL blood) weekly or biweekly
Check hematocrit/hemoglobin prior to each phlebotomy. Allow hematocrit/hemoglobin to fall by no more than 20% of prior level
Check serum ferritin level every 10-12 phlebotomies
Stop frequent phlebotomy when serum ferritin reaches 50-100 μg/L
Continue phlebotomy at intervals to keep serum ferritin between 50 and 100 μg/L
Avoid vitamin C supplements
Secondary iron overload due to dyserythropoiesis
Deferoxamine (Desferal) at a dose of 20-40 mg/kg body weight per day
Deferasirox (Exjade) given orally
Consider follow-up liver biopsy to ascertain adequacy of iron removal
Avoid vitamin C supplements

The decision to treat HH with phlebotomy is straightforward and easy to justify for patients with evidence of liver disease or other end-organ manifestations. The more difficult situation is the C282Y homozygote patient with a ferritin level of only 800 μg/L for example, with normal liver tests and no symptoms. Current longitudinal data are limited; some patients such as this will never progress to more serious problems and may not need to be treated. However, treatment is easy, safe, inexpensive, and could conceivably provide societal benefit (blood donation), and thus treatment is often initiated. Furthermore, there are no available, reliable indicators of who will develop complications. Conceivably, the rate of increase of serum ferritin will prove in the future to be an indicator of potential tissue and organ damage. In the absence of results from controlled trials, we currently favor proceeding to prophylactic phlebotomy in those individuals who tolerate and adhere to the regimen.

In those patients with advanced disease who may have cardiac arrhythmias or cardiomyopathy, there is an increased risk of sudden death with rapid mobilization of iron, most likely due to the presence of intracellular iron in a relatively toxic, low-molecular-weight chelate pool of iron. Pharmacological doses of vitamin C accelerate mobilization of iron to a level that may saturate circulating transferrin, resulting in an increase in pro-oxidant and/or free radical activity.^71^ Therefore, supplemental vitamin C should be avoided by iron-loaded patients, particularly those undergoing phlebotomy. No dietary adjustments are necessary, because the amount of iron absorption that an individual can affect with a low-iron diet is small (2-4 mg/day) compared to the amount mobilized with phlebotomy (250 mg/week). Reports of *Vibrio vulnificus* have been described in patients with HH who ingest raw shellfish; these foods should be avoided.^72^

Advanced cirrhosis is not reversed with iron removal, and the development of decompensated liver disease is an indication to consider orthotopic liver transplantation (OLT). In the past, survival of patients with HH who underwent liver transplantation was lower than in those who underwent liver transplantation for other causes of liver disease.^73^,^74^ Most posttransplantation deaths in patients with HH occurred in the perioperative period from either cardiac or infection-related^72^ complications.^75^ These complications were probably related to inadequate removal of excess iron stores before OLT. Currently, survival of patients with HH after OLT is comparable to other patients,^76^ at least in part because diagnosis and treatment occurs prior to OLT.

***Recommendations:***

***9. Patients with hemochromatosis and iron overload should undergo therapeutic phlebotomy weekly (as tolerated). (1A) Target levels of phlebotomy should be a ferritin level of 50-100 μg/L. (1B*)**

***10. In the absence of indicators suggestive of significant liver disease (ALT, AST elevation), C282Y homozygotes who have an elevated ferritin (but <1000 μg/L) should proceed to phlebotomy without a liver biopsy. (1B*)**

***11. Patients with end-organ damage due to iron overload should undergo regular phlebotomy to the same endpoints as indicated above. (1A*)**

***12. During treatment for HH, dietary adjustments are unnecessary. Vitamin C supplements and iron supplements should be avoided. (1C*)**

***13. Patients with hemochromatosis and iron overload should be monitored for reaccumulation of iron and undergo maintenance phlebotomy. (1A) Target levels of phlebotomy should be a ferritin level of 50-100 μg/L. (1B*)**

***14. We recommend treatment by phlebotomy of patients with non-HFE iron overload who have an elevated HIC. (1B*)**

## Treatment of Secondary Iron Overload

These guidelines have primarily concentrated on the management of HH, but it is reasonable to review the treatment of noninherited forms of secondary iron overload. The causes of secondary iron overload are listed in Table 3.

Phlebotomy is useful in certain forms of secondary iron overload (Table 8). Phlebotomy is clearly indicated in patients with PCT, and results in a reduction in skin manifestations. Total iron stores rarely exceeds 4-5 g. Secondary iron overload is sometimes seen in association with chronic hepatitis C, NAFLD, and ALD.^77^ There is no published evidence that phlebotomy is of benefit in ALD. In chronic hepatitis C, it has been shown that phlebotomy therapy reduces elevated ALT levels and achieves a marginal improvement in histopathology, but has no effect on virologic clearance.^78^ Currently, phlebotomy is not recommended for mild secondary iron overload (HIC < 2500 μg/g dry weight) in chronic hepatitis C. In NAFLD, studies have shown a benefit of therapeutic phlebotomy with improvement in parameters of insulin resistance and reduction in elevated ALT levels.^79^,^80^ Large-scale studies in patients with NAFLD have been proposed.

In secondary iron overload associated with ineffective erythropoiesis, iron chelation therapy with parenteral deferoxamine is the treatment of choice. Numerous studies have documented the efficacy of deferoxamine in preventing the complications of iron overload in β-thalassemia.^81^ Recently, deferasirox (Exjade), an orally administered iron-chelating drug, has been approved in the United States for treatment of secondary iron overload due to ineffective erythropoiesis. Studies are ongoing regarding its potential use in HH. However, recent concerns about complications have tempered enthusiasm for this drug in HH.^82^ Deferoxamine is usually administered by continuous subcutaneous infusion using a battery-operated infusion pump at a dose of 40 mg/kg/day for 8-12 hours nightly for 5-7 nights weekly. A total dose of approximately 2 g per 24 hours usually achieves maximal urinary iron excretion. Chelation therapy to reduce HIC < 15,000 μg/g dry weight significantly reduces the risk of clinical disease.^83^ The application of deferoxamine therapy is limited by cost, the need for a parenteral route of therapy, discomfort, inconvenience, and neurotoxcity. Monitoring iron reduction in patients with secondary iron overload is challenging. In contrast to HH, where serum ferritin reliably reflects iron burden during therapy, ferritin levels can be misleading in secondary iron overload. In some patients, it may be necessary to repeat liver biopsy to assess the progress of therapy and ensure adequate chelation.^84^ Monitoring 24-hour urinary iron excretion is sometimes helpful. By detecting magnetic susceptibility, a superconducting quantum interference device (SQUID) is capable of measuring HIC over a wide range, but this is a research technique that is available only in a few centers worldwide.^85^ The recent development of certain magnetic resonance imaging programs has shown promise in providing a noninvasive method to evaluate HIC.^86^,^87^ In patients with secondary iron overload, HIC provides an accurate quantitative means for monitoring iron balance.^88^

***Recommendations:***

***15. Iron chelation with either deferoxamine mesylate or deferasirox is recommended in iron overloaded patients with dyserythropoietic syndromes or chronic hemolytic anemia. (1A*)**

## Surveillance for Hepatocellular Cancer

In patients with HH who present with cirrhosis, the recent AASLD guidelines for HCC surveillance should be followed.^89^ These recommendations should be extended to patients with HH who have cirrhosis, whether they have had phlebotomy to restore normal iron levels. The relative risk for HCC is approximately 20, with an annual incidence of 3%-4%.^89^ Patients with HH with advanced fibrosis or cirrhosis should be screened regularly for HCC as per AASLD guidelines.

## General Population Screening

When considering the evidence required to determine whether general population screening should be performed for the C282Y mutation, a key factor is the clinical penetrance of C282Y homozygosity. Approximately 30% of C282Y homozygotes do not have phenotypic expression of excess iron stores in cross-sectional studies (Table 2). Because of this, from a public policy perspective, general population screening for HH is not indicated. In a large Norwegian study, 65,238 subjects were screened using TS, and when it was elevated on two determinations, cases were confirmed by genetic testing and/or liver biopsy.^11^ In 147 subjects, liver biopsies were performed and only four men and none of the women had cirrhosis (2.7% prevalence of cirrhosis). An Australian population study of 3011 individuals revealed 16 C282Y homozygotes. Of these 16, liver biopsy was performed in 11 cases with serum ferritin >300 μg/L, of whom three were identified with advanced fibrosis and one with cirrhosis who had associated ALD (6.3% prevalence of cirrhosis).^90^ Other prospective population studies have reached similar conclusions that the clinical penetrance of C282Y homozygosity is quite low.^10^,^12^ This discrepancy between the morbidity seen in referred patients and the lack of morbidity in screened patients is not unique to HH.

Economic models that have included genetic testing have suggested that population screening for HH would be effective if only 20% of patients developed life-threatening complications.^91^,^92^ The natural history of untreated HH has been illustrated in the Copenhagen Heart Study, where patients were followed for 25 years with serial ferritin testing without an awareness that they were C282Y homozygotes.^93^ Many patients did not demonstrate progression of iron overload as measured by serum ferritin, and the costs of investigating false positive iron tests in a screening program were considered significant. This has led some to consider that a genetic test should be done first, followed by measurement of serum ferritin. There have been concerns expressed about the adverse effects of genetic testing such as genetic discrimination; however, several studies have demonstrated that this is rarely a valid concern.^94^ Nonetheless, widespread population screening for HH is not recommended, whereas more selective screening in high-risk populations needs further study.^5^,^95^

***Recommendations:***

***16. Average risk population screening for HH is not recommended***.^93^ (***1B***)

## Screening for Non–*HFE*-related HH

The term “non–*HFE*-related HH” refers to several genetically distinct forms of inherited iron overload affecting individuals without *HFE* mutations.^17^ Several of the genes involved are hemojuvelin (*HJV*), ferroportin (*SLC40A1*), transferrin receptor 2 (*TFR2*), and hepcidin (*HAMP*). The non-*HFE* forms of inherited iron overload are rare, accounting for <5% of cases encountered, and genetic testing is largely unavailable except in research laboratories.

Screening for non–*HFE*-related HH is not recommended.

## Abbreviations

AASLD: American Association for the Study of Liver Diseases
ALD: alcoholic liver disease
ALT: alanine aminotransferase
AST: aspartate aminotransferase
BMP6: bone morphogenetic protein-6
C282Y: Cys282Tyr mutation
GRADE: Grading of Recommendation Assessment, Development, and Evaluation
H63D: His63Asp mutation
HAMP: hepcidin
HCC: hepatocellular carcinoma
HH: hereditary hemochromatosis
HIC: hepatic iron concentration
HII: hepatic iron index
HJV: hemojuvelin
OLT: orthotopic liver transplantation
PCT: porphyria cutanea tarda
S65C: Ser65Cys mutation
TfR2: transferrin receptor-2
TS: transferrin saturation
